# *Amanitaahmadii*, a new species of *Amanita* subgenus *Amanitina* section *Validae* from Pakistan

**DOI:** 10.3897/mycokeys.56.31819

**Published:** 2019-07-23

**Authors:** Sana Jabeen, Munazza Kiran, Junaid Khan, Ishtiaq Ahmad, Habib Ahmad, Hassan Sher, Abdul Nasir Khalid

**Affiliations:** 1 Department of Botany, Division of Science and Technology, University of Education, Lahore, Punjab, Pakistan University of Education Lahore Pakistan; 2 Department of Botany, University of the Punjab, Quaid-e-Azam Campus-54590, Lahore, Punjab, Pakistan University of the Punjab Lahore Pakistan; 3 Center for Plant Sciences and Biodiversity, University of Swat, Swat, Khyber Pakhtunkhwa, Pakistan University of Swat Swat Pakistan; 4 Centre of Plant Biodiversity and Conservation, University of Peshawar, Peshawar, Khyber Pakhtunkhwa, Pakistan Islamia College University Peshawar Pakistan; 5 Islamia College University, Peshawar, Khyber Pakhtunkhwa, Pakistan University of Peshawar Peshawar Pakistan

**Keywords:** Amanitaceae, nrDNA, Swat

## Abstract

A new species from coniferous forests in Pakistan, *Amanitaahmadii*, is described on the basis of morpho-anatomy and molecular data set analyses. This species is characterized by its medium-sized to large basidiomata, grayish brown to brown pileal surface and rimose pileus margin with gray to dark brown verrucose veil remnants, a cream stipe with bulbous base having grayish brown or brown longitudinal striations above the annulus, a scaly surface towards the base, globose to broadly ellipsoid and amyloid basidiospores, and the absence of clamped septa in all tissues. Molecular phylogenetic analyses based on ITS and LSU sequences confirmed its identity as a new taxon nested within subgen. Amanitinasect.Validae.

## Introduction

Amanitaceae E. J. Gilbert is a large family of agaricoid fungi that has been classified by many mycologists and split into various genera subgenera and sections (Corner and Bas 1962; Bas 1969). During recent years, it has been split into two genera, *Amanita* Pers., a genus of putatively ectomycorrhizal fungi, and *Saproamanita* Redhead, Vizzini, Drehmel & Contu, a genus of putatively saprotrophic fungi (Redhead et al. 2016). This generic split has been rejected by Tulloss et al. (2016) based in part on the guidelines of Vellinga et al. (2015) for introducing new genera. Concise amended characterizations have been provided for the monophyletic family Amanitaceae and its two monophyletic genera, *Amanita* and *Limacella* Earle. This declaration is based on the current use of next-generation sequencing in studies of fungal ecology opposing the splitting of the genus. Recently Cui et al. (2018) and Yang et al. (2018) inferred the phylogeny of Amanitaceae based on multi-locus sequencing data. The results indicated that Amanitaceae is monophyletic and consists of five genera. The genus *Amanita* consists of 95% of the species which are characterized by agaricoid basidiomata, colorless and hyaline, ballistosporic and smooth basidiospores, free lamellae, presence of volval remnants (Persoon 1797). A total of 540 known species of *Amanita* are distributed worldwide (Yang 2000, Kirk et al. 2008, Menolli et al. 2009, Tulloss 2009, Wartchow et al. 2009, Justo et al. 2010, Wartchow and Gamboa-Trujillo 2012, Cho et al. 2015, Hosen et al. 2015, Tang et al. 2015, Wartchow and Cortez 2016, Jabeen et al. 2017, Cui et al. 2018, Kiran et al. 2018a, b). From Pakistan, 19 species of *Amanita* are known to date (Ahmad et al. 1997, Jabeen et al. 2017, Kiran et al. 2018a, b). Tulloss et al. (2001) described one new species, *A.pakistanica* Tulloss, S.H. Iqbal & Khalid, but refrained from describing two more due to lack of materials. The work on these species is in progress by several workers, and it is estimated that the total number of *Amanita* from Pakistan could be above 50. Many taxa of the genus have been reported as edibles (Tulloss and Bhandary 1992, Buyck 1994, Montoya-Esquivel 1997), though some others are deadly poisonous (Yang 2015, Cai et al. 2016). Most of the species are ecologically important forming mycorrhizal symbiosis (Yang 1997, 2000, Kiran et al. 2018a).

Members of Amanitasubgen.Amanitina (E. J. Gilbert) E. J. Gilbert have non-striated pileus margins, attenuate lamellulae and amyloid basidiospores (Cui et al. 2018)). Six sections in this subgenus are recognized (Cui et al. 2018), based on the morphology of the remnants of the universal veil and the pileal margin. The sect. Validae is characterized by pilei that are usually distinctly colored, margins that are non-appendiculate and do not exceed the gill margin, non-fragile and membranous annuli and basal bulbs that are usually small (Tulloss and Yang 2018, Yang 1997, Cui et al. 2018).

During our ongoing studies of ectomycorrhizal fungi in Khyber Pakhtunkhwa province, we collected specimens of an unknown *Amanita* species belonging to Amanitasubgen.Amanitinasect.Validae. The aim of the present study was to characterize and identify the taxon based on molecular phylogeny using the sequence data of the internal transcribed spacer (ITS) and partial large subunit (LSU) of ribosomal RNA. Here, we describe this taxon as a new species.

## Materials and methods

### Sampling sites

Specimens were collected from three different areas in two districts of Khyber Pakhtunkhwa province of Pakistan. One of these, the Swat district, has a very rich biodiversity. The mountains are covered with snow throughout the winter and in summer temperature ranges between 16–33 °C. The average annual precipitation in Swat district ranges from 1000 mm to 1200 mm. The first area, Gabin Jabba, is a lush green valley in Swat district, which is characterized by a moist temperate vegetation with *Piceasmithiana* (Wall.) Boiss. and *Abiespindrow* Royle as the dominant tree species. Mashkun, the second area in Swat district, is in the western part of the Himalayas. This collection site is a dry temperate forest with *A.pindrow*, *P.smithiana* and *Cedrusdeodara* (Roxb. ex D. Don) G. Don as the dominant tree species along with *Pinuswallichiana* A. B. Jacks.

The third area is Kumrat valley, which lies at the extreme North of the Dir Upper district. It is located in the foothills of the Hindu Kush mountains with an elevation of about 950–2440 m (Siddiqui et al. 2013). Snowfall occurs frequently in winter, rainfall during monsoon season ranges from 100 mm to 255 mm. Forests are dominated by a mixture of *C.deodara*, *A.pindrow*, *Piceasmithiana*, and *Pinuswallichiana*, and *Populusnigra* L. is the main broad-leaved tree.

### Macroscopic and microscopic characterization

Specimens were collected during routine macrofungal surveys and photographed in their natural habitats using a Nikon D3200 camera. Morphological features of fresh specimens were recorded and colors were designated using Munsell Soil Color Charts (Munsell 1975) and then forced-air dried for long term preservation. For detailed anatomical descriptions, tissues from different parts of the basidiomata were mounted on glass slides in 5% Potassium Hydroxide solution (KOH; w/v). Phloxine (1% w/v aqueous solution) was used for a better contrast. Melzer’s reagent was used to check the amyloidity of basidiospores. Anatomical features were noted under a compound microscope (MX4300H, Meiji Techno Co., Ltd, Japan). Measurements were recorded using a Carl Zeiss (Jena) ocular micrometer and line drawings were made using Leitz Wetzlar camera lucida. Size and shape of basidiospores are presented in a form following the description of ranges for biometric variables according to Tulloss (2016). Voucher specimens are deposited in the Herbarium at the University of the Punjab (LAH), Quaid-e-Azam Campus, Lahore, Pakistan and at the Swat University Herbarium (SWAT), Swat, Pakistan.

### DNA extraction, PCR and sequencing

For genomic DNA extraction, a standard CTAB method (Bruns 1995) was followed. Internal transcribed spacer regions along with central 5.8S region of nuclear ribosomal DNA (nrDNA) were amplified (Gardes and Bruns 1993) using forward primer ITS1F and reverse primer ITS4 (White et al. 1990). For LSU amplification, LR0R as forward and LR5 as reverse primers were used (Ge et al. 2014). The PCR products were sent to Macrogen Inc. (Korea) for sequencing.

### Sequence alignment and phylogenetic analyses

Consensus sequences were generated from the sequences obtained by both primers (forward and reverse) in BioEdit software v. 7.2.5 (Hall 1999). Sequences of Amanitasubgen.Amanitinasect.Validae at NCBI (http://www.ncbi.nlm.nih.gov/) and from published literature (Kim et al. 2013, Cai et al. 2014, Cui et al. 2018) were added to the datasets. Taxa from the sect. Phalloideae were chosen as outgroup (Cui et al. 2018). Shorter ITS and LSU sequences were omitted from the final matrices. Species and specimens used for the molecular phylogenetic analyses are given in Table 1. Multiple sequences were aligned using online webPRANK by EMBL-EBI, Wellcome Trust Genome Campus, UK (https://www.ebi.ac.uk/goldman-srv/webprank/). The phylogeny was inferred by maximum likelihood (ML) analysis using model selection for best DNA analysis for each dataset in MEGA6 software (Tamura et al. 2013). Models with the lowest BIC scores (Bayesian Information Criterion) were considered to describe the substitution pattern the best. Non-uniformity of evolutionary rates among sites may be modeled by using a discrete gamma distribution (+G) with 5 rate categories and by assuming that a certain fraction of sites are evolutionarily invariable (+I). The phylogenetic analyses were performed at 1000 bootstrap replicates. Percentage identity and divergence in nrDNA-ITS of the taxa were analyzed using MegAlign (DNAStar, Inc.). Sequences generated in this study were submitted to GenBank under accession numbers KY996724, KY996755, MF116158 and MF070490 for ITS and KY996725 and MK166021 for LSU.

**Table 1.** Species and specimens of *Amanita* used for the molecular phylogenetic analyses.

Species	Voucher	Country	GenBank accession number		Reference
			ITS	LSU	
A. aff. brunnescens	BW_HF 10C	USA	–	HQ539661	–
A. aff. citrina	BW_PNC	USA	–	HQ539662	–
	HKAS 34170	China	AY436449	AY436489	Zhang et al. 2004, Thongbai et al. 2016
A. aff. flavorubens	PSMCC 121	USA	–	HQ539663	–
	BW_HF-FR	USA	–	HQ539664	–
A. aff. fritillaria	HKAS56832	China	KJ466372	KJ466479	Cai et al. 2014, Thongbai et al. 2016
	HKAS57649	China	KJ466373	KJ466480	Cai et al. 2014
A. aff. spissacea	2C5	Japan	AB973749	–	–
* A. ahmadii *	LAH35010	Pakistan	KY996724	KY996725	–
	SWAT0001351	Pakistan	MF070490	–	–
	LAH35241	Pakistan	KY996755	MK166021	–
	LAH35242	Pakistan	MF116158	–	–
* A. augusta *	DBB49390	USA	JQ937287		–
	DBB21873	USA	JX515564	–	–
*A.augusta* as “*A.franchetii*”	07040	USA	GQ250398	–	–
* A. bisporigera *	RET 377-9	USA	KJ466374	KJ466434	Thongbai et al. 2016
* A. brunneolocularis *	ANDES_F313 NVE57	Colombia	FJ890033	FJ890044	Vargas et al. 2011
* A. brunnescens *	RET 637-7	USA	KT006762	KT006766	Thongbai et al. 2016
	BW_HP12	USA	–	HQ539674	–
	RET 529-10	USA	KP284273	KP284284	–
	RET 554-1	USA	KP284275	KP284285	–
	RET 549-9	USA	–	KP284283	–
	JS94/2	–	–	AF097379	Drehmel et al. 1999
* A. castanea *	MFLU 15-1424	Thailand	KU904823	KU877539	Thongbai et al. 2016
A. cf. flavorubescens	JMP0098	USA	EU819454	–	Palmer et al. 2008
A. cf. spissacea	BZ2015-40	Thailand	KY747464	–	Cai et al. 2012
	OR1214	Thailand	KY747469	KY747478	Cai et al. 2012
* A. citrina *	LEM 960298	Japan	AB015679	–	Oda et al. 1999, Thongbai et al. 2016
	JM96/61	–	–	AF097378	–
	TM02_102	Canada	–	EU522722	Porter et al. 2008
	KA12-1226	South Korea	KF245908	KF245892	Kim et al. 2013
	JSH s.n.	–	–	AF041547	–
	JS94/1	–	–	AF097377	Drehmel et al. 1999
	ANDES_F405 IP25	Colombia	–	FJ890046	Vargas et al. 2011
	BW JLR 102106-1	USA	–	HQ539679	–
	KA12-1612	South Korea	KF245909	KF245893	Kim et al. 2013
* A. citrinoindusiata *	HKAS100522	China	MH508320	MH486468	Cui et al. 2018
	HKAS58884	China	MH508323	MH486471	Cui et al. 2018
	HKAS58886	China	MH508324	MH486472	Cui et al. 2018
	HKAS58796	China	MH508321	MH486469	Cui et al. 2018
	HKAS58888	China	MH508325	MH486473	Cui et al. 2018
	HKAS58874	China	MH508322	MH486470	Cui et al. 2018
* A. excelsa *	HKAS 31510	Germany	AY436453	AY436491	Thongbai et al. 2016
	Ge 816	China	–	HQ539691	–
* A. flavipes *	KA12-0685	South Korea	KF245911	KF245895	Kim et al. 2013
	HKAS 36582	China	AY436455		Zhang et al. 2004
	KA12-1517	South Korea	KF245912	KF245896	Kim et al. 2013
* A. flavoconia *	TENN61564	USA	JF313655	–	–
	BW_PH22	–	–	HQ539693	–
	ANDESF408CV3	Colombia	FJ890029	FJ890041	Thongbai et al. 2016
	TM03_435 25S	Canada	–	EU522816	Porter et al. 2008
	NVE 351	Colombia	KF937301	–	Vasco-Palacios et al. 2014
	NVE 242	Colombia	KF937300	–	Vasco-Palacios et al. 2014
* A. flavoconia *	HKAS 34047	USA	AY436456		Zhang et al. 2004
	RV5Aug96	–	–	AF042609	Moncalvo et al. 2000
* A. flavorubens *	RET 295-9	USA	–	HQ539694	–
* A. flavorubescens *	TENN61660	USA	JF313650	–	–
	F:PRL6062	USA	GQ166902	–	Thongbai et al. 2016
	RV96/102	–	–	AF097380	Drehmel et al. 1999
* A. franchetii *	JM96/27	–	–	AF097381	Drehmel et al. 1999
A.franchetiif.lactella as “*A.franchetii*”	DBBJUS01	Spain	JX515563	–	–
	DBB52095	Bulgaria	JX515562	–	–
	DBB51482	Bulgaria	JX515561	–	–
A.franchetiif.queletii as “*A.aspera*”	IFO-8262	–	AF085485	–	Lim and Jung 1998
* A. fritillaria *	–	China	JF273505	–	Legendre et al. 2009
	HKAS 38331	China	AY436457	–	Zhang et al. 2004
	KA12-1231	South Korea	KF245913	KF245897	Kim et al. 2013
* A. lavendula *	RET 639-7	USA	KP866163	KR865979	Thongbai et al. 2016
* A. luteofusca *	PSC 1093b	Australia	–	HQ539705	–
* A. luteolovelata *	PSC 2187	Australia	–	HQ539706	–
* A. morrisii *	RET 672-6	USA	KR919762	KR919770	–
	RET 271-7	USA	KT213441	KT213442	Thongbai et al. 2016
	RET 445-10	USA	KR919760	KR919768	–
* A. novinupta *	GO-2009-234	Mexico	KC152066	–	–
	GO-2009-315	Mexico	KC152065	–	–
	GO-2009-301	Mexico	KC152067	–	–
	RET 060-2	USA	KF561974	KF561978	Thongbai et al. 2016
	RET 093-10	USA	–	HQ539716	–
	NY 00066710	USA	KJ535437	KJ535441	–
* A. phalloides *	GDGM:40312	Italy	KC755034	–	
* A. porphyria *	LEM960303	Japan	AB015677	–	Oda et al. 1999
	DAVFP:26784	USA	JF899548	–	
	RET 079-1	Switzerland	KP866181	KP866192	Thongbai et al. 2016
	HKAS 31531	China	AY436471	AY436500	Thongbai et al. 2016
	RET 309-8	Norway	KP866176	KP866189	–
	RET 404-2	Czech Republic	KP866171	KP866184	–
	RET 404-9	Czech Republic	–	KP866185	–
* A. rubescens *	JMP0003	USA	EU819464	–	Palmer et al. 2008
	TRTC156957	Canada	JN020972	–	Dentinger et al. 2011
	LE241998	Russia	JF313652	–	–
	RK01-01	Denmark	AJ889923	–	–
	EMF4	China	JF273507	–	–
	LEM950063	Japan	AB015682	–	Oda et al. 1999
	ASIS23255	South Korea	KM052530	–	–
	ASIS23444	South Korea	KM052535	–	–
	KA 12-1221	Korea	KF245919	KF245903	Thongbai et al. 2016
	RET 122-8	Turkey	–	HQ539735	–
	ANDES_F416 NVE160	Colombia	FJ890031	FJ890043	Vargas et al. 2011
	RV5Aug96	–	–	AF042607	Moncalvo et al. 2000
	RV97/23	–	–	AF097383	Drehmel et al. 1999
	JM96/53	–	–	AF097382	Drehmel et al. 1999
	KA12-0936	South Korea	KF245918	KF245902	Kim et al. 2013
*A.* sp.	ANDES_F241 IP24	Colombia	FJ890032	FJ890047	Vargas et al. 2011
	RET 516-10	USA	KP711830	KP711838	–
	RET 516-5	USA	KP711836	KP711837	–
	RET 530-1	USA	KT072736	KT072737	–
	RET 539-8	USA	KT072735	KT072738	–
	HKAS 38419	China	AY436474	AY436502	Thongbai et al. 2016
* A. spissa *	UP541	–	EF493270	–	Nygren et al. 2008
	KF02-47	–	AJ889924	–	–
	UP542	–	EF493271	–	Nygren et al. 2008
	KA12-0884	South Korea	KF245910	KF245894	Kim et al. 2013
	NYBG 47779	Germany	–	HQ539743	–
* A. spissacea *	LEM960187	Japan	AB015683	–	Oda et al. 1999
	ASIS24872	South Korea	KM052552	KU139485	–
	ASIS26240	–	KT894841	KU139454	–
	ASIS24978	–	KM052550	KU139487	–
	ASIS24775	–	KM052543	KU139484	–
	ASIS24949	–	KM052546	KU139486	–
* A. virosa *	HKAS 56694	China	JX998030	JX998058	Cai et al. 2012
	HMJAU23304	China	KJ466431	KJ466498	Cai et al. 2012
	JM 97/42	–	–	AF159086	Moncalvo et al. 2000

## Results

### Phylogeny

Consensus sequences of the ITS region were BLAST searched at NCBI. These sequences showed 98% identity to A.aff.fritillaria (KJ466372 and KJ466373) sequences from China (Cai et al. 2014) with 94–100% query cover. It also showed 95% identity with an *A.franchetii* (JX515561) sequence from Bulgaria with 100% query cover and 0.0 E value. The LSU consensus sequence BLAST at NCBI showed 99% identity to A.aff.flavoconia (HQ539663) and *Amanita* sp. (KT072738) sequences from the eastern USA and *A.fritillaria* (KF245897) sequences from South Korea with 99% query cover.

**Figure 1.**
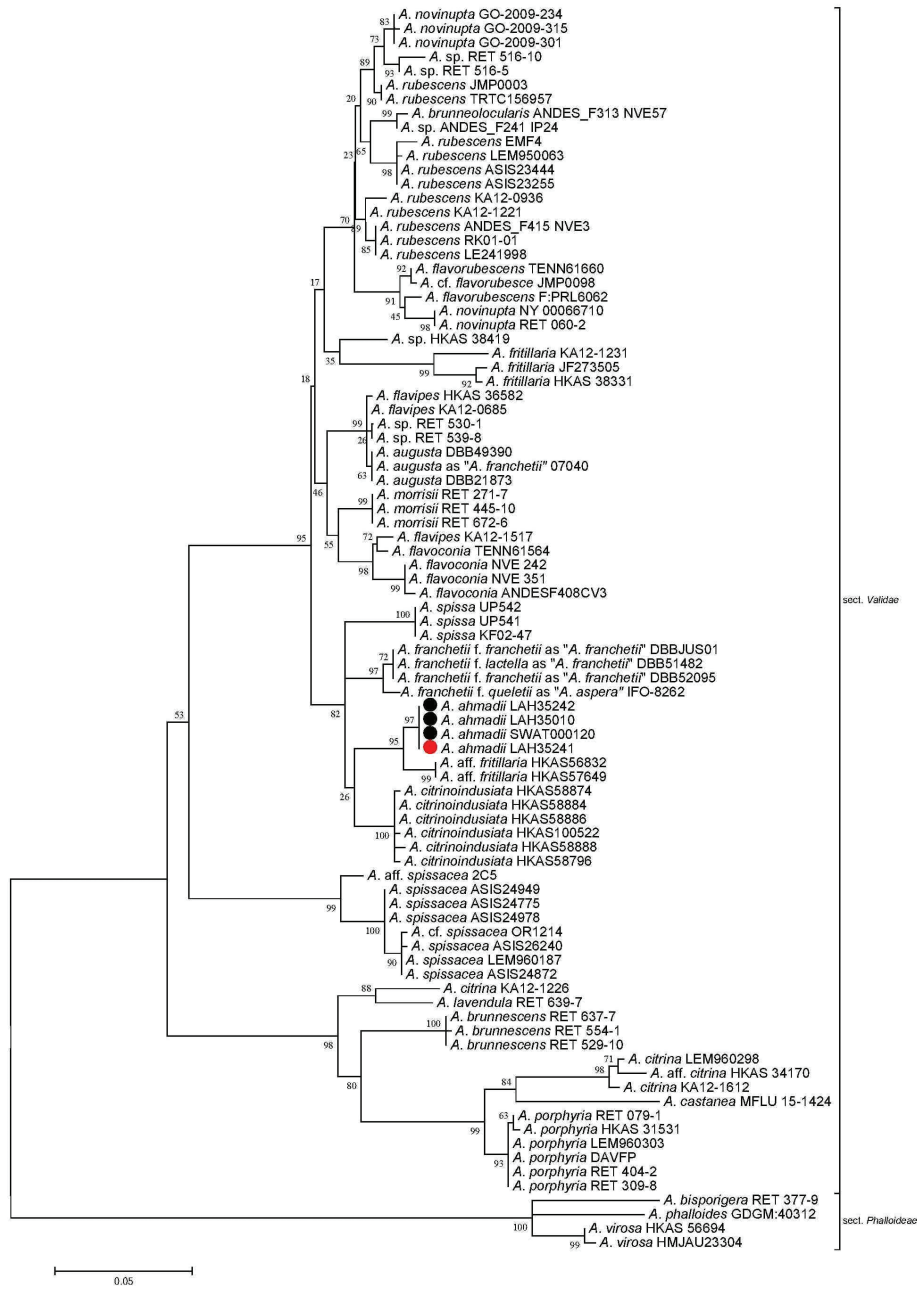
Molecular phylogenetic analysis of ITS sequences using the maximum likelihood method based on the Tamura 3-parameter model (Tamura 1992). The percentage of trees in which the associated taxa clustered together is shown next to the branches. A discrete gamma distribution was used to model evolutionary rate differences among sites (5 categories (+*G*, parameter = 0.4454)). The tree is drawn to scale, with branch lengths measured in the number of substitutions per site. The analysis involved 88 nucleotide sequences. There were a total of 1018 positions in the final dataset. Sequences generated during the present investigation are marked with bullets. Red represents the holotype.

**Figure 2.**
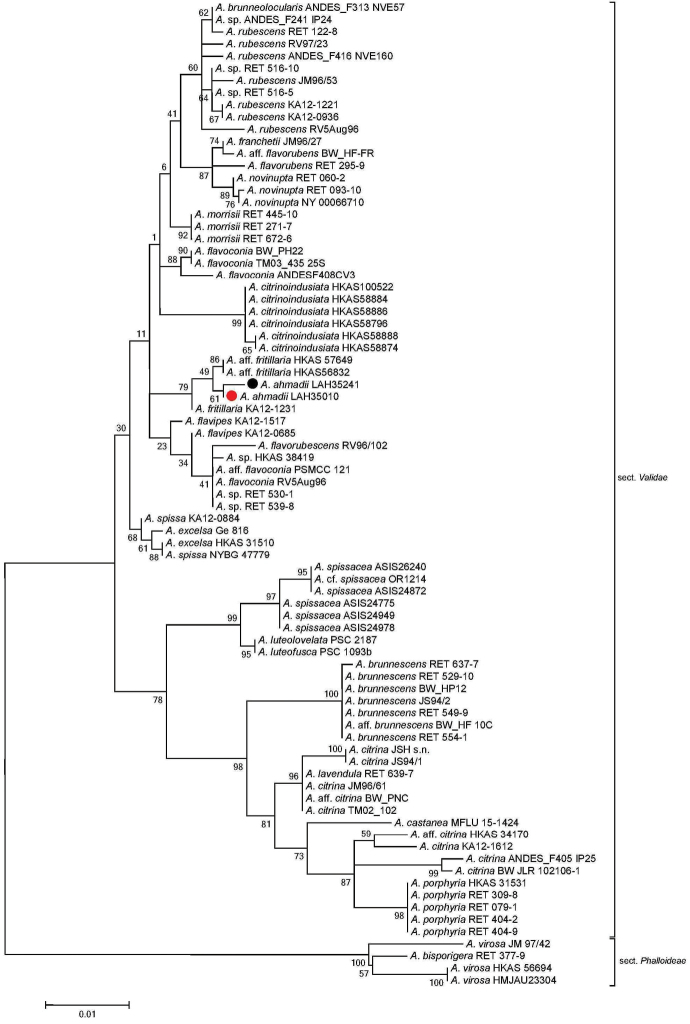
Molecular phylogenetic analysis of LSU sequences by using the maximum likelihood method based on the Kimura 2-parameter model (Kimura 1980). A discrete gamma distribution was used to model evolutionary rate differences among sites (5 categories (+*G*, parameter = 0.2164)). The tree is drawn to scale, with branch lengths measured in the number of substitutions per site. The analysis involved 81 nucleotide sequences. There were a total of 871 positions in the final dataset. Sequences generated during the present investigation are marked with bullets. Red represents the holotype.

**Figure 3.**
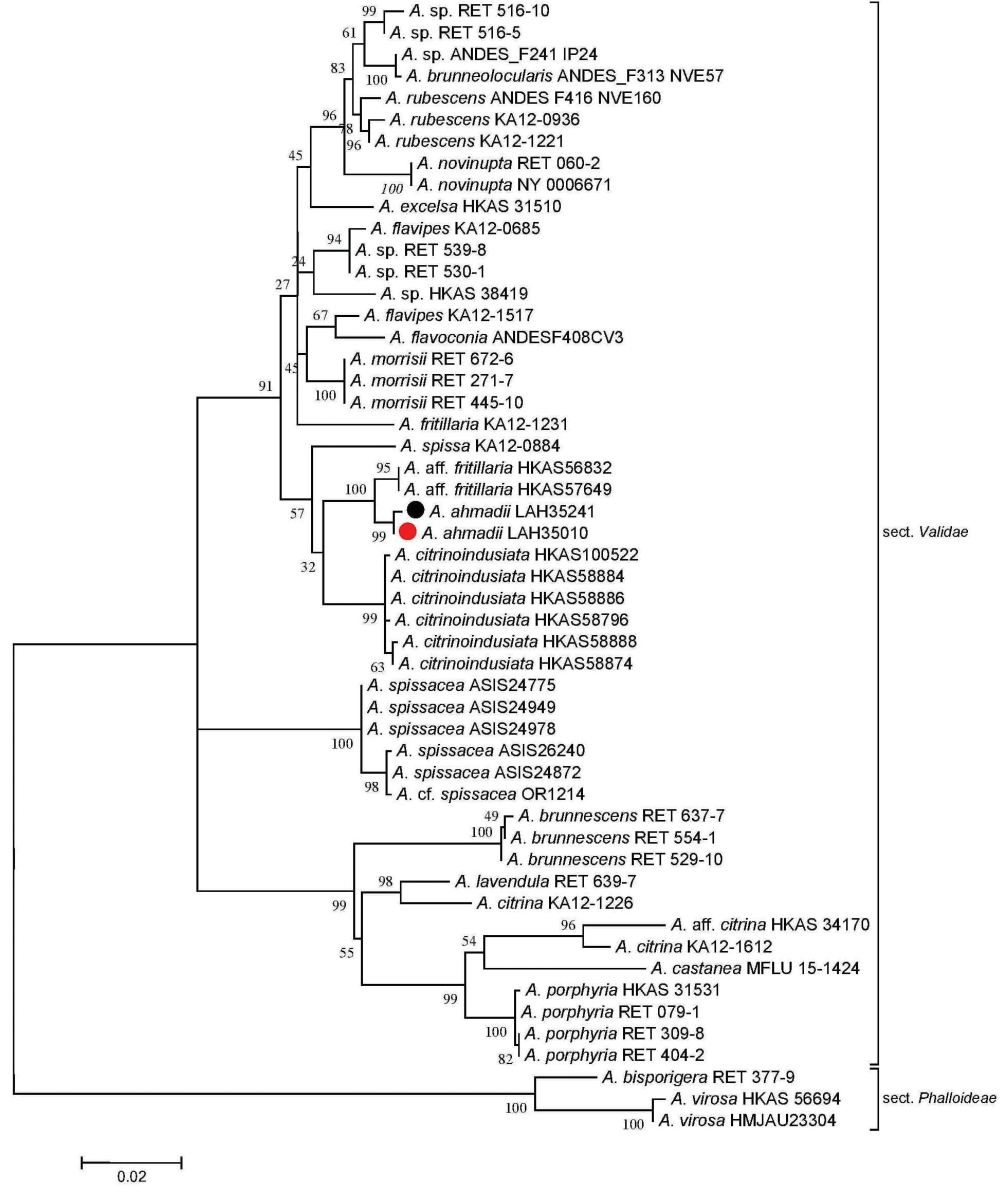
Molecular phylogenetic analysis of ITS+LSU sequences by using the maximum likelihood method based on the Tamura-Nei model (Tamura and Nei 1993). A discrete gamma distribution was used to model evolutionary rate differences among sites (5 categories (+*G*, parameter = 0.2250)). The rate variation model allowed for some sites to be evolutionarily invariable ([+*I*], 43.3848% sites). The tree is drawn to scale, with branch lengths measured in the number of substitutions per site. The analysis involved 52 nucleotide sequences. There were a total of 1760 positions in the final dataset. Sequences generated during the present investigation are marked with bullets. Red represents the holotype.

Taxa from subgen. Amanitinasect.Phalloideae (Fr.) Quél. were chosen as the outgroup (Kim et al. 2013). The sequences generated during this study clustered with the similar taxa in sect. Validae (Figs 1–3). Our species clustered with A.aff.fritillaria, *A.citrinoinduciata*, A.franchetiif.franchetii , A.franchetiif.lactella (as *A.franchetii* in GenBank), A.franchetiif.queletii (as *A.aspera* in GenBank) and *A.spissa* in phylogenetic analysis. However, *A.ahmadii* separated from A.aff.fritillaria with a strong bootstrap value of 95%, 49% and 100% in ITS, LSU and ITS+LSU sequence dataset analyses, respectively (Figs 1–3).

### Taxonomy

#### Amanita ahmadii

Taxon classificationFungiAgaricalesAmanitaceae

Jabeen, I. Ahmad, Kiran, J. Khan & Khalid sp. nov.

2952bed1-8c25-54a4-bc10-394d36e002bc

MB821204

Figs 4 , 5

##### Diagnosis.

Small to medium-sized basidiomata, grayish brown to brown pileal surface having rimose and non-appendiculate pileal margins, verrucose, gray to dark bluish or brown veil remnants, dry and split stipe surface at the base forming scales, globose to subglobose, smooth, amyloid basidiospores.

##### Holotype.

Pakistan, Khyber Pakhtunkhwa province, Malakand division, Swat district, Mashkun, 2500 m a.s.l., on soil under *Cedrusdeodara*, 5 Sept. 2013, Sana Jabeen SJ35 (LAH35010; GenBank ITS: KY996724; LSU: KY996725).

##### Etymology.

The species epithet *ahmadii* refers to Sultan Ahmad, the pioneer Pakistani mycologist.

##### Description.

Pileus 4–7 cm in diameter, convex to flat at maturity; cuticle gray (2.5BG4/2) to grayish brown (10YR3/2) or brown (2.5Y4/4) with time; surface dry; universal veil remnants on pileus verrucose, aligned in one direction, scattered, gray (2.5Y4/2) to dark brown (2.5Y2/2); margins non-appendiculate, incurved when young, highly rimose by maturity. Lamellae off-white (2.5BG4/2) to cream (5Y9/4) becoming brownish when dry, adnexed, subdistant to close; edges entire. Lamellulae small (1/3 of the lamellae), attenuate, truncate. Stipe 6.7–9 × 0.6–1.5 cm, apex slightly wider and white, with up to 1.5 cm wide bulbous base, central, cylindrical; surface with grayish brown (5GY5/2) striations above the annulus, splitting towards the base forming scales on white (2.5BG4/2) to cream (5Y9/4) context. Annulus superior, membranous, skirt-like, with longitudinal striations on the upper surface, gray (2.5Y4/2) with a darker lower part. Universal veil absent. Ordorless and not changing color upon bruising.

Basidiospores [60/3/3] (6.5) 7–8.5 (9.5) × (6) 6.5–7.5 (8) µm, Q = (1) 1.03–1.22 (1.33), avg Q = 1.10, globose to broadly ellipsoid, amyloid in Melzer’s reagent. Basidia (32) 34.5–59 (67) × 7–8 µm, clavate, frequently 4 sterigmate, 2 sterigmata also observed, thin-walled, hyaline in 5% KOH. Subhymenium pseudoparenchymatous, cells isodiameteric, intermixed and densely packed. Veil remnants made up of hyphae with terminal subglobose to elongated cells (42.5) 49.5–54 (57) × (13) 13–16 (19) µm on a branched filament 3–4 µm wide; septa frequent; clamp connections absent. Pileipellis filamentous, 4–5 µm in diameter, branched, septate; clamp connections absent, light brown with some hyaline tissue in 5% KOH. Universal veil remnants of globose to subglobose cells (6.8) 8–12.2 (12.7) × (4.4) 7.5–10.5 (11) µm with filaments (0.7) 0.9–2.6 (3.5) µm in diameter. Hyphae from stipe 3–24 µm wide, filamentous, branched, hyaline in 5% KOH, septate; clamp connections absent in all tissues.

##### Habitat and distribution.

In coniferous forests of Pakistan with a moist temperate to dry temperate climate.

##### Additional specimens examined.

Pakistan, Khyber Pakhtunkhwa province, Malakand division, Dir Upper district, Kumrat, 2232 m a.s.l., on soil under conifers, 2 Sept. 2015, Abdul Nasir Khalid FS82 (LAH35241; GenBank ITS: KY996755; LSU: MK166021); Swat district, Mashkun, 2500 m a.s.l., on soil under *Cedrusdeodara*, 4 Aug. 2013, Ishtiaq Ahmad IS213P65 (LAH35242; GenBank ITS: MF116158); Gabin Jabba valley, 2450 m a.s.l., on soil under *Piceasmithiana*, 30 Aug. 2015, Junaid Khan GJ-1508 (SWAT0001351; GenBank ITS: MF070490).

**Figure 4.**
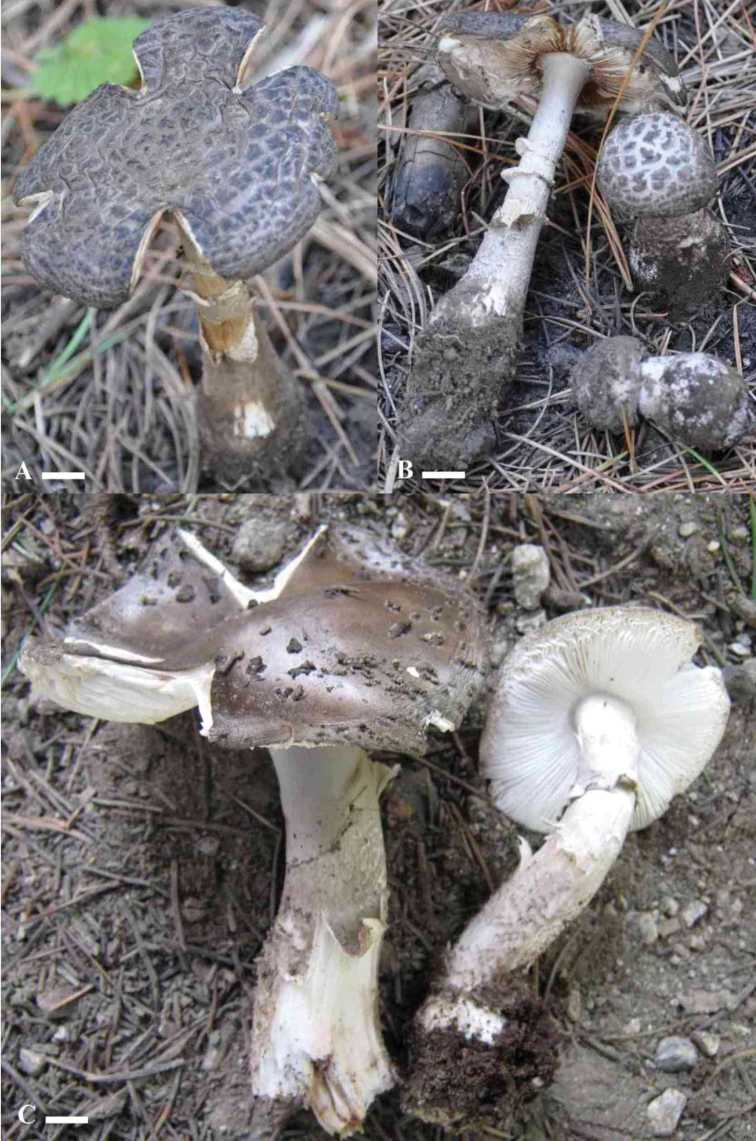
*Amanitaahmadii* basidiomata. **A, B**LAH35010 (holotype) **C** SWAT0001351. Photos by Abdul Nasir Khalid and Junaid Khan. Scale bars: 1 cm (**A**); 1.2 cm (**B**); 0.5 cm (**C**).

**Figure 5.**
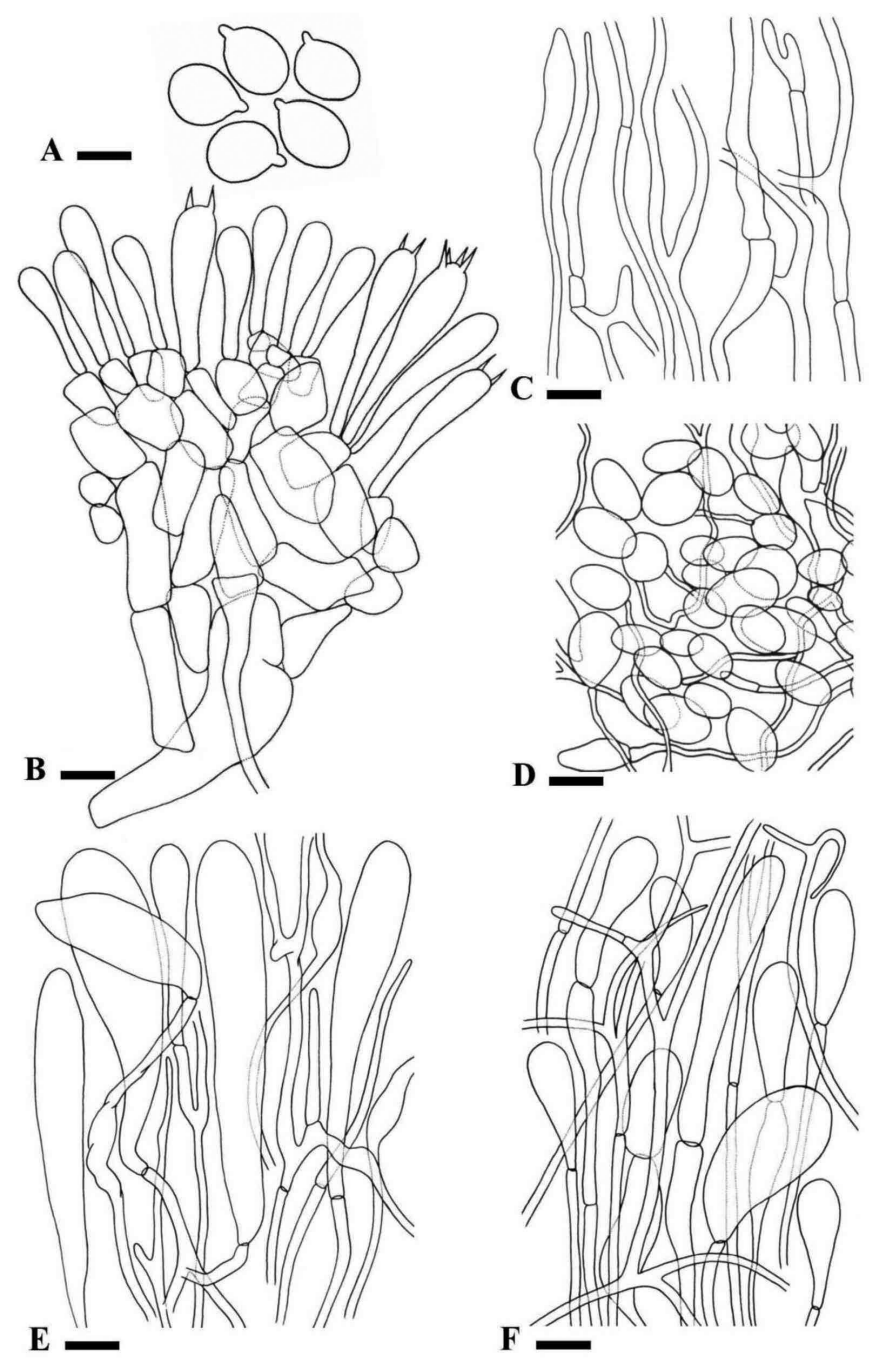
*Amanitaahmadii*LAH35010 (holotype). **A** Basidiospores **B** Basidia, basidioles and subhymenium **C** Pileipellis **D** Universal veil remnants on pileus surface **E** Hyphae from stipe **F** Partial veil. Drawings by Sana Jabeen. Scale bars: 5.5 µm (**A**); 8 µm (**B–D**); 22 µm (**E, F**).

## Discussion

*Amanitaahmadii* is characterized by its grayish brown to brown pileus surface with abundant gray to dark brown verrucose veil remnants and by its rimose margins. Anatomically it is characterized by its globose to broadly ellipsoid basidiospores. The species is morphologically similar to *A.fritillaria* Sacc. by its grayish to brownish gray pileus surface, and verrucose volval remnants. *Amanitafritillaria* differs by bearing ellipsoid basidiospores (Corner and Bas 1962, Yang 1997, 2005, 2015). In phylogenetic trees based on ITS, LSU and combined sequence datasets of both regions, *A.fritillaria* was inferred as a distinct lineage from *A.ahmadii*.

Amanitaaff.fritillaria (HKAS56832 and HKAS57649, Cai et al. 2014) forms a sister clade to *A.ahmadii* (Figs 1–3), but it is morphologically distinct. The former taxon possesses a brownish and purplish pileus surface (Zhu L. Yang pers. comm.) while the latter has a grayish brown or brown pileus surface with highly rimose margins (Cai et al. 2014). Amanitafritillariaf.malayensis Corner & Bas was described from Singapore (Corner and Bas 1962), but more recently was also found in subtropical, evergreen, broad-leaved forests in China; it differs from *A.ahmadii* in having a dark umber to rather pale grayish umber pileus (Yang 2005, 2015).

The European sequences labeled as “*A.franchetii*” and “*A.aspera*” in GenBank are close relatives of *A.ahmadii* in the ITS phylogenetic analysis. *Amanitafranchetii* (Boud.) Fayod is somewhat variable in appearance and there are three morphological infraspecific taxa, including A.franchetiif.franchetii (Boud.) Fayod (JX515562 and JX515563), A.franchetiif.lactella Neville & Poumarat (JX515561) and A.franchetiif.queletii (Bon & Dennis) Neville & Poumarat (AF085485) (Neville and Poumarat 2004). The last taxon most closely resembles *A.ahmadii* but differs in having more yellow hues on the stipe and pronounced reddening on the bulb with age. *Amanitaaugusta* Bojantchev & R. M. Davis, as “*A.franchetii*” in GenBank (GQ250398), another species from western North America looks similar to *A.ahmadii* but its yellowish brown pileus with yellow universal veil remnants and ellipsoid spores (Bojantchev and Davis 2013) distinguishes it from *A.ahmadii*. During phylogenetic analyses, all these taxa were inferred as distinct species.

The novel species also showed differences from *A.castanea* Thongbai, Tulloss, Raspé & K. D. Hyde from Thailand. *Amanitacastanea* bears a viscid, shiny and sericeous pileal surface, which is dark brown at center and light brown to brownish orange towards margin, with universal veil mostly towards the margin, rarely over disc, as scattered gray to brownish gray, reddish brown to grayish brown warts or small floccose patches and globose basidiospores (Thongbai et al. 2016). All these characters distinguish *A.castanea* from *A.ahmadii*. In molecular phylogenetic analyses, *A.castanea* is clustered with the species in a distant clade within sect. Validae (Figs 1–3). *Amanitaahmadii* also showed morphological distinctions from *A.citrinoindusiata* Zhu L. Yang, Y. Y. Cui & Q. Cai, a newly reported species in the same section from China. This species is characterized by its robust, brownish gray, gray to dark gray pileus and a stipe bearing a citrine to yellowish annulus. This suggests it is a separate species from *A.ahmadii* (Cui et al. 2018). Molecular data also supports the separation of these two taxa in phylogenetic trees (Figs 1–3).

The European *A.excelsa* Gonn. & Rabenh is also morphologically close to *A.ahmadii* in having a gray-brown pileus. However, *A.excelsa* differs from *A.ahmadii* in having mealy, gray irregular and non-persistent patches of volval remnants on the pileus. The volva in *A.excelsa* has 2–5 pale ochre brown zones of friable material above the bulb, and lastly, the broadly ellipsoid to ellipsoid, occasionally elongate basidiospores also distinguish *A.excelsa* from *A.ahmadii* (Neville & Poumarat, 2004). The phylogenetic position of these taxa also indicates that they are separate. Based on morphological characters and molecular phylogenic analysis, our new species belongs to Amanitasubgen.Amanitinasect.Validae.

## Supplementary Material

XML Treatment for Amanita ahmadii
